# The m^6^A reader MhYTP2 regulates the stability of its target mRNAs contributing to low nitrogen tolerance in apple (*Malus domestica*)

**DOI:** 10.1093/hr/uhad094

**Published:** 2023-05-04

**Authors:** Tianli Guo, Zehua Yang, Ru Bao, Xiaomin Fu, Na Wang, Changhai Liu, Fengwang Ma

**Affiliations:** State Key Laboratory of Crop Stress Biology for Arid Areas/Shaanxi Key Laboratory of Apple, College of Horticulture, Northwest A&F University, Yangling, Shaanxi 712100, China; State Key Laboratory of Crop Stress Biology for Arid Areas/Shaanxi Key Laboratory of Apple, College of Horticulture, Northwest A&F University, Yangling, Shaanxi 712100, China; State Key Laboratory of Crop Stress Biology for Arid Areas/Shaanxi Key Laboratory of Apple, College of Horticulture, Northwest A&F University, Yangling, Shaanxi 712100, China; State Key Laboratory of Crop Stress Biology for Arid Areas/Shaanxi Key Laboratory of Apple, College of Horticulture, Northwest A&F University, Yangling, Shaanxi 712100, China; State Key Laboratory of Crop Stress Biology for Arid Areas/Shaanxi Key Laboratory of Apple, College of Horticulture, Northwest A&F University, Yangling, Shaanxi 712100, China; State Key Laboratory of Crop Stress Biology for Arid Areas/Shaanxi Key Laboratory of Apple, College of Horticulture, Northwest A&F University, Yangling, Shaanxi 712100, China; State Key Laboratory of Crop Stress Biology for Arid Areas/Shaanxi Key Laboratory of Apple, College of Horticulture, Northwest A&F University, Yangling, Shaanxi 712100, China

## Abstract

Studies have shown that the m^6^A reader primarily affects genes expression by participating in the regulation of mRNA localization, splicing, degradation, translation, and other metabolic processes. Previously, we discovered that the apple (*Malus domestica*) m^6^A reader MhYTP2 bound with and destabilized m^6^A-modified *MdMLO19* mRNA. In addition, it enhanced the translation efficiency of m^6^A-modified mRNA of *MdGDH1L*, encoding a glutamate dehydrogenase, which confers resistance to powdery mildew. In this study, we report the function of MhYTP2 in the regulation of resistance to low nitrogen (N). The overexpression of *MhYTP2* enhances the resistance of apple to low N. We show that MhYTP2 binds with and stabilizes the mRNAs of *MdALN*, which participates in the allantoin catabolic process and cellular response to N starvation in apple; *MdPIDL*, which participates in root hair elongation; *MdTTG1*, which is involved in the differentiation process of trichomes; and *MdATG8A*, which is a core participant in the regulation of autophagy. In addition, MhYTP2 accelerates the degradation of *MdRHD3* mRNA, which regulates root development. RNA immunoprecipitation-seq and electrophoretic mobility shift assays show that the mRNAs of *MdALN*, *MdATG8A*, *MdPIDL*, *MdTTG1*, and *MdRHD3* are the direct targets of MhYTP2. Overexpressing or knocking down the above genes in *MhYTP2* overexpressing plants dismisses the function of MhYTP2 under low N, suggesting the role of MhYTP2 is dependent on those genes. Together, these results demonstrate that MhYTP2 enhances the resistance of apple to N deficiency by affecting the stability of the bound mRNAs.

## Introduction

N^6^-methyladenosine (m^6^A) is the most common post-transcriptional modification in eukaryotic mRNA that has been detected in many species [1]. A series of mammalian m^6^A methylase (writers) complex subunits, demethylase (erasers), and m^6^A-binding proteins (readers) have been identified, suggesting that m^6^A affects mRNA translation, alternative splicing, nuclear transport, and mRNA decay among others and subsequently affects multiple biological processes [1–9]. The dynamically reversible process of m^6^A is regulated by writers and erasers. The readers recognize the m^6^A modifications and determine the fate of RNA [10–12]. The YT512-B Homology (YTH) domain is a recognized m^6^A binding domain [13, 14]. Several human YTH domain family proteins have been shown to be m^6^A readers [15–17]. There are 13 YTH domain proteins in *Arabidopsis* [18]. Among them, ECT2 has been characterized as an m^6^A reader [19–21], and redundantly regulate leaf growth and organogenesis with ECT3 and ECT4 [19, 22]. ECT2 has been shown to bind with the m^6^A-modified mRNA and controls its stability, thereby controlling trichome morphology [20, 21]. A *Malus hupehensis* YTH domain that contains RNA binding protein 2, MhYTP2, participates in multiple biological processes including leaf senescence and fruit ripening [13, 23]. Under drought conditions, MhYTP2 enhances the efficiency of water use in apple (*Malus domestica*) by activating abscisic acid (ABA) and ethylene signaling [24]. Recently, we showed that MhYTP2 is an m^6^A reader. MhYTP2 regulates the powdery mildew resistance of apple by binding with and destabilizing the *MdMLO19* and *MdMLO19-X1* mRNAs and enhancing the translation efficiency of antioxidant genes [25].

Nitrogen (N) is a crucial nutrient element affecting plant biological processes [26]. Plants are often faced with low N availability under natural agricultural conditions [27]. N deficiency reduces the rate of plant cell division, photosynthesis, and leaf growth [28], thus, affecting plant growth and therefore reducing yield and commodity value. MhYTP2 has been shown to function in some biological processes. However, the function and regulatory mechanism of MhYTP2 in response to N deficiency remains unclear.

Plants use complex mechanisms to deal with N deficiency, including many physiological, molecular, and cellular adaptations. Plants accumulate allantoin, which helps to mobilize N in plants [29]. *Arabidopsis* plants with allantoin to allantoate gene (*aln*) mutations have improved resistance to abiotic stress [30, 31]. Root structure and root hairs growth and elongation are major factors determining the location and volume of exploited soil [32]. PID kinase participates in root geotropism regulation in *Arabidopsis* roots [33]. Overexpress *PID* reduced gravitropic response in the roots of transgenic plants [34]. In addition, The TTG1-bHLH-MYB complex activates trichome initiation and patterning [35]. Studies have also shown that RHD3 participates in adventitious root and lateral root formation and root hair development regulation [15]. Autophagy is also reported to participate in plants’ low N response. Apple plants elevate their autophagic activity, starch degradation, and speed of sugar metabolism to enhance low N resistance [36]. The apple autophagy-related gene *MdATG8i* transcripts were induced in response to N depletion [37].

**Figure 1:**
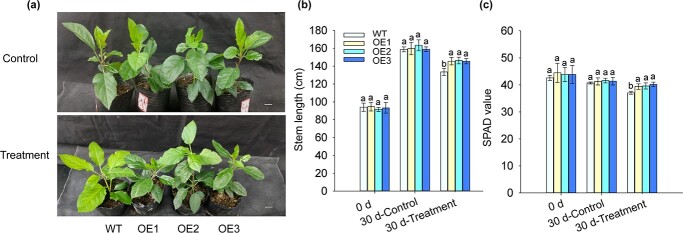
Overexpression of *MhYTP2* enhanced the tolerance of apple to nitrogen deficiency. **a** Phenotypes of the WT and *MhYTP2* overexpressing plants in response to nitrogen deficiency treatment. Scale bar, 1 cm. **b** Stem lengths and **c** SPAD values of the WT and *MhYTP2* overexpressing plants. Data represent the means ± SD from three replicates. Different lowercase letters indicate the statistically significant differences among the WT and *MhYTP2* overexpressing plants of different treatments.

**Figure 2:**
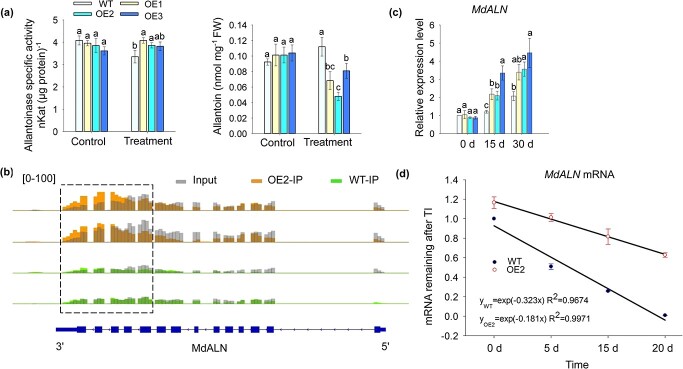
Overexpression of *MhYTP2* increased the activity of allantoinase in response to nitrogen deficiency. **a** The allantoinase activity and allantoin concentration in *MhYTP2* overexpressing and WT plants under different nitrate conditions. FW, fresh weight. **b** The levels of modification of *MdALN* by m^6^A in OE2 and the WT plants. **c** The expression of *MdALN* in *MhYTP2* overexpressing and WT plants on Day 0, 15, and 30 of treatment. **d** The mRNA duration of *MdALN* in OE2 and WT plants. Data represent the means ± SD from three replicates. Different lowercase letters indicate the statistically significant differences among the WT and *MhYTP2* overexpressing plants of different treatments.

**Figure 3:**
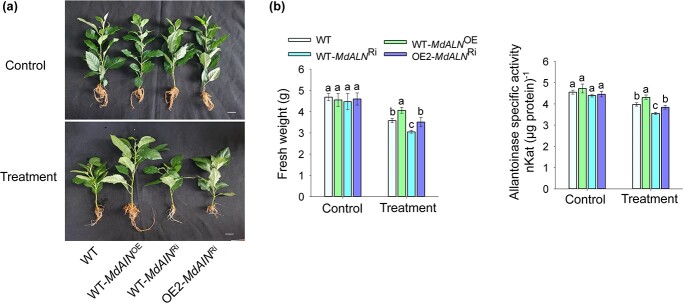
Overexpression of *MdALN* increased the resistance of apple to nitrogen deficiency. **a** Phenotypes of the WT, WT-*MdAIN*^OE^*,* WT-*MdAIN*^Ri^, and OE2-*MdAIN*^Ri^ in response to N deficiency treatment. Scale bar, 1 cm. **b** The fresh weight and allantoinase activity of the WT, WT-*MdAIN*^OE^*,* WT-*MdAIN*^Ri^, and OE2-*MdAIN*^Ri^ plants. Data represent the means ± SD from three replicates. Different lowercase letters indicate the statistically significant differences among the WT and *MhYTP2* overexpressing plants of different treatments.

Genetic regulation networks, such as the post-transcriptional regulation, can be a powerful strategy for stress responses [38]. However, to our knowledge, these mechanisms remain limited. Therefore, elucidating whether an m^6^A reader MhYTP2 affects the stability of mRNAs related to N metabolism and signaling will help us understand the function of m^6^A in plant regulation of low N. In the study, the m^6^A modifications on several mRNAs of important genes that are involved in N signaling are bound and regulated by MhYTP2, which contributes to a better performance of *MhYTP2* overexpressing plants than the wild type (WT) when the plants suffered from N deficiency.

## Results

### Overexpression of *MhYTP2* enhanced the tolerance of apple to N deficiency

Three previously obtained *MhYTP2* overexpressing apple lines OE1, OE2, and OE3 (the genetic background is GL3, a variant of ‘Gala’) [24] were used to further examine the function of *MhYTP2* under a limited supply of N. As shown in Fig. 1a, Under normal growth condition, plants overexpressing *MhYTP2* showed no significant phenotypic differences from WT plants. However, under N deficient condition, *MhYTP2* overexpressing plants had significantly longer stem lengths (9.1–9.6% increase) and higher SPAD values (6.2–8.4% increase), indicating the total chlorophyll content, than WT (Fig. 1b and c).

### Overexpression of *MhYTP2* increased the activity of allantoinase in response to N deficiency

Under normal growth conditions, allantoinase activity and allantoin concentration of *MhYTP2* overexpressing plants were not significantly different from WT. After the N had been depleted for 30 days, allantoinase activity of *MhYTP2* overexpressing plants was significantly increased (14.3–21.8% increase) compared with WT, while allantoin concentration was significantly decreased in the *MhYTP2* overexpressing plants, ranging from 0.44 to 0.74 times relative to that observed in WT plants (Fig. 2a). The previous m^6^A-seq data [25] indicated that the levels of modification of m^6^A of *M. domestica* allantoinase-like gene (*MdALN*) increased significantly at the 3′ untranslated region (UTR) and exon regions in *MhYTP2* overexpressing line OE2 compared with WT (Fig. 2b). To confirm that *MdALN* mRNA is the target of MhYTP2, an RNA electrophoretic mobility shift assay (EMSA) was conducted. The result showed that MhYTP2 directly bound with the *MdALN* mRNA that had been modified with m^6^A (Fig. S1, see online supplementary material). We then examined the level of expression of *MdALN* that encodes allantoinase. *MdALN* was more highly expressed in *MhYTP2* overexpressing lines than WT under N starvation condition (Fig. 2c). Considering the proposed functions of MhYTP2, we hypothesized that MhYTP2 could bind with and affect the stability of *MdALN* transcripts. We then measured the durations of *MdALN* transcripts, which showed that the *MdALN* transcript was degraded more slowly in OE2 compared with WT plants (Fig. 2d).

### Overexpression of *MdALN* increased the resistance of apple to N deficiency

To investigate the biological function of *MdALN* in N deficiency, we obtained transgenic apple roots in which *MdALN* was overexpressed and knocked down*,* namely WT-*MdAIN*^OE^ and WT-*MdAIN*^Ri^ (WT indicates that the plants used to induce hairy roots were GL3), respectively (Fig. S2, see online supplementary material), using the hairy roots transgenic system [39]. In addition, to explore the possibility that empty vector pCambia2300 (2300) or pK7GWIWG2D (pK7) affects the growth of seedlings or the expression of target genes, we also obtained the plants WT-2300 and WT-pK7 that expressed only empty vectors. The expressed empty vectors were shown to have no effect on the growth of seedlings or the expression of target genes under both normal growing and N starvation condition (Fig. S3, see online supplementary material). As shown in Fig. 3a, the WT, WT-*MdAIN*^OE^, and WT-*MdAIN*^Ri^ had no significant differences in the fresh weight and allantoinase activity under normal growing conditions. However, after being treated with N deficiency for 30 days, there were significant differences in biomass and allantoinase activity between WT and plants with *MdAIN* overexpressed or knocked-down in roots (Fig. 3b). These results indicate that *MdALN* positively regulates the response of apples to N deficiency.

Considering that the *MdALN* transcript was relatively stabilized in OE2 than WT (Fig. 2d), we knocked down *MdALN* in OE2 (OE2-*MdAIN*^Ri^) (Fig. S2, see online supplementary material) to investigate whether the MhYTP2 function in regulate low N resistance requires *MdALN*. As shown in Fig. 3b, *MhYTP2* overexpressing plants with *MdALN* knocked down behaved similarly as WT, suggesting that the MhYTP2 function in regulating low N resistance requires *MdALN*.

### Overexpression of *MhYTP2* changed the root architecture by affecting the mRNA stability of genes related to root development in response to N deficiency

**Figure 4:**
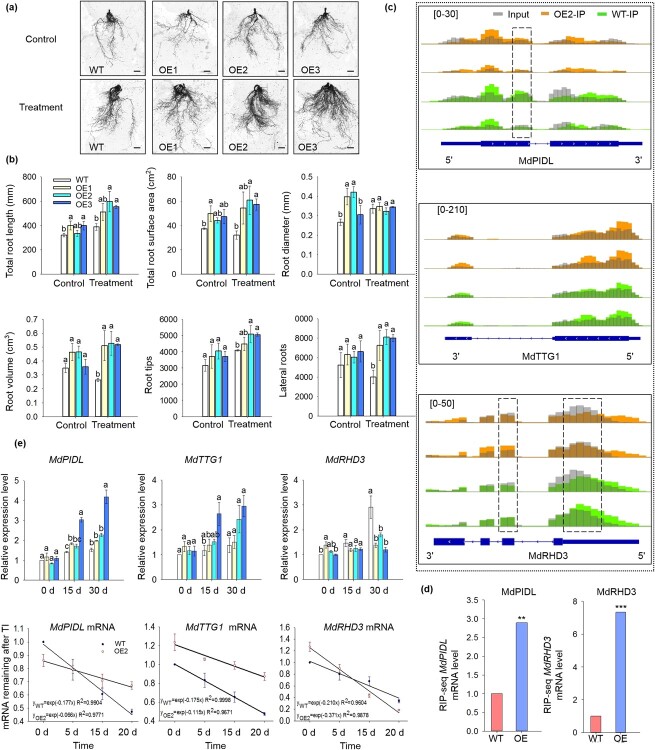
Overexpression of *MhYTP2* changed the root architecture by affecting the mRNA stability of genes related to root development in response to nitrogen deficiency. **a** Phenotypes of WT and *MhYTP2* overexpressing plant roots under different nitrate conditions. Scale bar, 1 cm. **b** Root system architecture in the plants grown under different nitrate conditions. **c** The levels of *MdPIDL*, *MdTTG1*, and *MdRHD3* mRNAs modified by m^6^A in OE2 and WT plants. **d** Validations of the MhYTP2 binding with m^6^A modified *MdPIDL* and *MdRHD3* mRNAs by RIP-seq. **e** The expression of *MdPIDL*, *MdTTG1*, and *MdRHD3* in *MhYTP2* overexpressing and WT plants on Day 0, 15, and 30 of treatment and mRNA duration of *MdPIDL*, *MdTTG1*, and *MdRHD3* in the transgenic line OE2 and WT plants. Data represent the means ± SD from three replicates. Different lowercase letters indicate the statistically significant differences among the WT and *MhYTP2* overexpressing plants of different treatments. ^**^Significant differences at *P* < 0.01. ^***^Significant differences at *P* < 0.001.

The root growth of *MhYTP2* overexpressing plants was better than that of WT plants under N deficiency conditions (Fig. 4a). Under normal growing conditions, the *MhYTP2* overexpressing plants had an increase in the aspect of total root length, surface area, and diameter, than the WT plants. The changes in volume, numbers of root tips and laterals did not differ between *MhYTP2* overexpressing and WT roots. Under N starvation conditions, the total root length, surface area, volume, number of root tips and forks of *MhYTP2* overexpressing plants significantly increased than WT, while the changes in diameters were not significant (Fig. 4b). To explore why the root growth was regulated in *MhYTP2* overexpressing plants, we carefully examined our previous m^6^A-seq data and found that the mRNAs of some genes related to root development, such as *M. domestica* protein kinase PINOID-like gene (*MdPIDL*)*, M. domestica* protein TRANSPARENT TESTA GLABRA 1-like gene (*MdTTG1*)*,* and *M. domestica* protein ROOT HAIR DEFECTIVE 3 homolog gene (*MdRHD3*)*,* contain m^6^A sites [25]. In particular, compared with WT, the levels of m^6^A that modified *MdPIDL* decreased in exon 1 of 2 in OE2; *MdTTG1* was slightly increased in the entire mRNA in OE2, and the modification of *MdRHD3* by m^6^A was in the 5'UTR in WT plant but in exon 2 of 4 in OE2 (Fig. 4c). As shown in Fig. S1 (see online supplementary material), MhYTP2 bound with mRNAs that had been modified by m^6^A. The mRNAs of *MdPIDL, MdTTG1,* and *MdRHD3* all contain m^6^A modifications, suggesting that they are direct targets for MhYTP2. In addition, an RNA immunoprecipitation (RIP)-seq experiment was conducted, which showed that MhYTP2 bound with the mRNAs of *MdPIDL* and *MdRHD3* [25] (Fig. 4d). We then measured the expression levels of the transcripts in *MhYTP2* overexpressing and WT plants. The *MdPIDL and MdTTG1* expression levels were higher in *MhYTP2* overexpressing plants than WT under N starvation conditions, while the *MdRHD3* expression level was lower in *MhYTP2* overexpressing plants than WT under N starvation conditions (Fig. 4e). We hypothesized that the m^6^A binding function of MhYTP2 affects the stability of the transcripts of *MdPIDL, MdTTG1,* and *MdRHD3*. We then performed the transcription inhibition assays to measure their duration, which showed that the *MdPIDL* and *MdTTG1* transcripts were degraded more slowly in OE2 than WT, particularly for the mRNA of *MdPIDL.* In contrast, the *MdRHD3* transcripts were degraded more rapidly in OE2 than WT (Fig. 4e)*.*

### *MdPIDL*, *MdTTG1*, and *MdRHD3* regulate the resistance of apple to N deficiency

**Figure 5:**
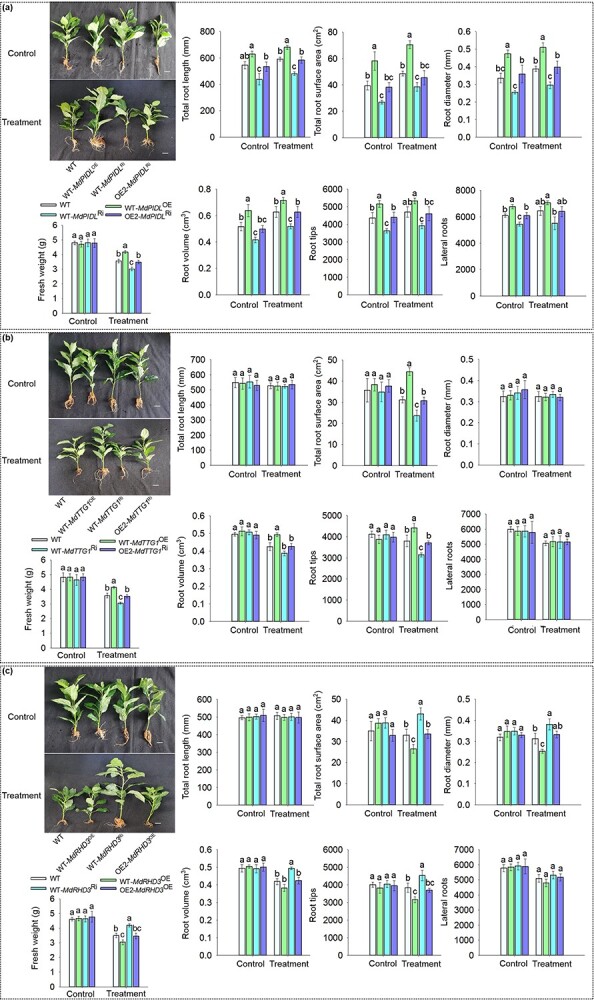
*MdPIDL*, *MdTTG1* and *MdRHD3* regulate the resistance of apple to nitrogen deficiency. **a** Root system architecture and fresh weight in the WT, WT-*MdPIDL*^OE^*,* WT-*MdPIDL*^Ri^, and OE2-*MdPIDL*^Ri^ plants grown under different nitrate conditions. **b** Root system architecture and fresh weight in the WT, WT-*MdTTG1*^OE^*,* WT-*MdTTG1*^Ri^, and OE2-*MdTTG1*^Ri^ plants grown under different nitrate conditions. **c** Root system architecture and fresh weight in the WT, WT-*MdRHD3*^OE^*,* WT-*MdRHD3*^Ri^, and OE2-*MdRHD3*^OE^ plants grown under different nitrate conditions. Scale bar, 1 cm. Data represent the means ± SD from three replicates. Different lowercase letters indicate the statistically significant differences among the WT and *MhYTP2* overexpressing plants of different treatments.

To investigate the biological function of *MdPIDL*, *MdTTG1,* and *MdRHD3* in N deficiency, we obtained plants with transgenic hairy roots, including WT-*MdPIDL*^OE^, WT-*MdPIDL*^Ri^, WT-*MdTTG1*^OE^, WT-*MdTTG1*^Ri^, WT-*MdRHD3*^OE^, and WT-*MdRHD3*^Ri^ (Fig. S2, see online supplementary material). We found that *MdPIDL* increased the low N resistance of apple based on the measurement of fresh weight (Fig. 5a). Further analysis found that *MdPIDL* promoted root growth (Fig. 5a). Under N starvation condition, the overexpression of *MdTTG1* increased the surface area and the numbers of root tips (Fig. 5b). Moreover, the overexpression of *MdRHD3* reduced the surface area of roots, diameter, and numbers of root tips under N starvation conditions (Fig. 5c).

Because the *MdPIDL* and *MdTTG1* transcripts were degraded more slowly in OE2 than WT (Fig. 4e), we knocked down *MdPIDL* and *MdTTG1* in OE2 (OE2-*MdPIDL*^Ri^ and OE2-*MdTTG1*^Ri^) (Fig. S2, see online supplementary material). The biomass production and root structure parameters did not differ significantly between OE2-*MdPIDL*^Ri^ and WT, as well as between OE2-*MdTTG1*^Ri^ and WT, suggesting that the MhYTP2 regulated the resistance to N deficiency in apple through *MdPIDL* and *MdTTG1* (Fig. 5a and b)*. MdRHD3* transcripts were degraded more rapidly in OE2 than WT (Fig. 4e). When *MdRHD3* was overexpressed in OE2 (OE2-*MdRHD3*^OE^) (Fig. S2, see online supplementary material), no significant difference was found between OE2-*MdRHD3*^OE^ and WT in terms of the biomass production and root structure parameters, indicating that the function of MhYTP2 in regulating the resistance of apple to low N was through *MdRHD3* (Fig. 5c)*.* These results suggest that MhYTP2 contributes to the low N resistance in apple by regulating *MdPIDL*, *MdTTG1*, and *MdRHD3* at the posttranscriptional level.

### Overexpression of *MhYTP2* stabilized the *MdATG8A* mRNA for an autophagy-related protein

**Figure 6:**
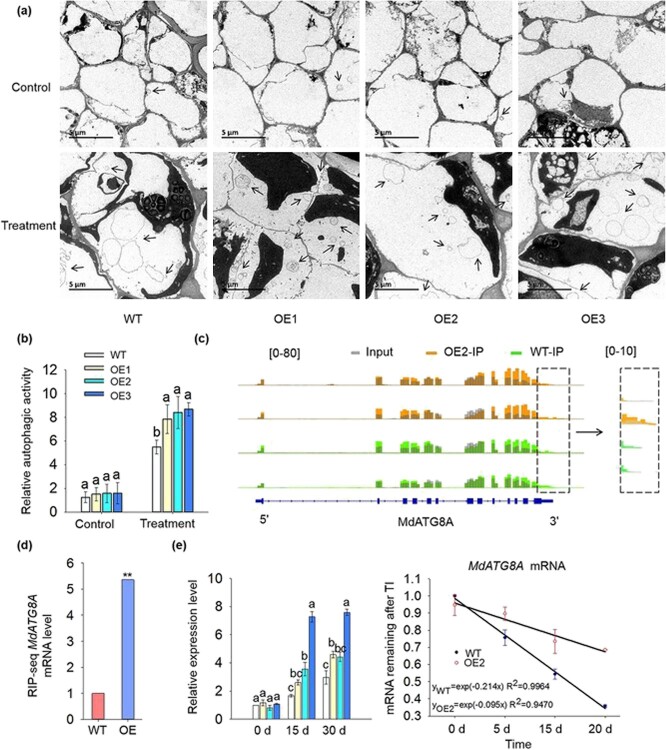
Overexpression of *MhYTP2* stabilizes the *MdATG8A* mRNA for an autophagy-related protein. **a** Representative images of autophagic structures in root cells from WT and *MhYTP2* overexpressing lines on day 30 of treatment. Scale bar, 5 μm. **b** Relative autophagic activity normalized to the activity of WT or *MhYTP2* overexpressing lines shown in **a**. **c** The levels of modification of *MdATG8A* mRNA by m^6^A in OE2 and WT plants. **d** Validations of the MhYTP2 binding with m^6^A modified *MdATG8A* mRNA by RIP-seq. **e** The expression of *MdATG8A* in *MhYTP2* overexpressing and WT plants on Day 0, 15, and 30 of treatment and the mRNA duration of *MdATG8A* in OE2 and WT. Data represent the means ± SD from three replicates. Different lowercase letters indicate the statistically significant differences among the WT and *MhYTP2* overexpressing plants of different treatments. ^**^Significant differences at *P* < 0.01.

The roots contained very few autophagosome structures in *MhYTP2* overexpressing and WT leaves under normal growth condition (Fig. 6a). Thirty days of N starvation condition treatment stimulated more autophagosomes in *MhYTP2* overexpressing plants than those of WT. The autophagy occurrence and relative autophagy activity of apple were significantly enhanced (42.9–57.6% increase) by the overexpression of *MhYTP2* when the plants suffered from N deficiency (Fig. 6a and b).

We further explored the mechanism that underlies the phenotype of increased resistance to N starvation of *MhYTP2* overexpressing plants. The m^6^A-seq data [25] showed that the autophagy-related gene *MdATG8A* mRNA contained m^6^A sites in their 3'UTR. The levels of modification of *MdATG8A* by m^6^A were significantly increased in OE2 compared with WT (Fig. 6c). The RIP-seq results also showed that MhYTP2 can bind with the m^6^A-containing *MdATG8A* mRNA [25] (Fig. 6d). We then determined the expression of *MdATG8A* in *MhYTP2* overexpressing and WT plants and found that level of expression of *MdATG8A* was higher in *MhYTP2* overexpressing plants when the plants suffered from N deficiency (Fig. 6e). We measured the duration of *MdATG8A* transcript, which showed that the *MdATG8A* transcript was degraded more rapidly in WT compared with OE2 plants (Fig. 6e).

### *MdATG8A* overexpression increased the resistance of apple to N deficiency

**Figure 7:**
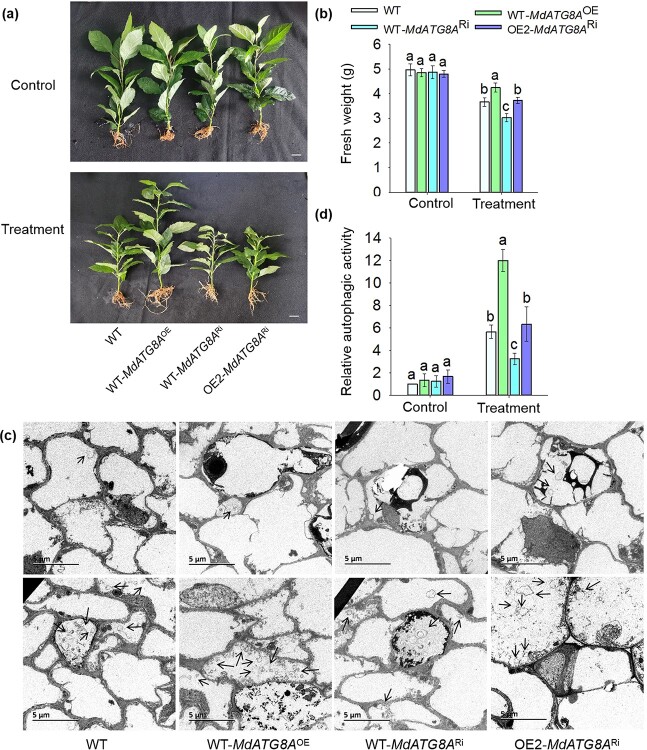
*MdATG8A* overexpression increased the resistance of apple to nitrogen deficiency. **a** Phenotypes of the WT, WT-*MdATG8A*^OE^, WT-*MdATG8A*^Ri^, and OE2-*MdATG8A*^Ri^ in response to deficiency treatment. Scale bar, 1 cm. **b** The fresh weight of the WT, WT-*MdATG8A*^OE^, WT-*MdATG8A*^Ri^, and OE2-*MdATG8A*^Ri^ plants. **c** Representative images of autophagic structures in the root cells from WT, WT-*MdATG8A*^OE^, WT-*MdATG8A*^Ri^, and OE2-*MdATG8A*^Ri^ plants on day 30 of treatment. Scale bar, 5 μm. **d** The relative autophagic activity of the WT, WT-*MdATG8A*^OE^, WT-*MdATG8A*^Ri^, and OE2-*MdATG8A*^Ri^ plants. Data represent the means ± SD from three replicates. Different lowercase letters indicate the statistically significant differences among the WT and *MhYTP2* overexpressing plants of different treatments.

To investigate the biological function of *MdATG8A* in N deficiency, we obtained plants with transgenic roots that overexpressed or knocked down *MdATG8A,* designated WT-*MdATG8A*^OE^ and WT-*MdATG8A*^Ri^, respectively (Fig. S2, see online supplementary material) [39]. Under normal growing conditions, the WT, WT-*MdATG8A*^OE^, and WT-*MdATG8A*^Ri^ had no significant differences in the fresh weight and relative autophagic activity. After being treated with N deficiency for 30 days, the WT-*MdATG8A*^OE^ plants accumulated more autophagosomes, produced more biomass, and displayed relatively higher autophagic activity than WT. In contrast, the WT-*MdATG8A*^Ri^ roots accumulated fewer autophagosomes, produced less biomass, and had relatively lower autophagic activity than WT (Fig. 7b, c, and d), suggesting that *MdATG8A* positively regulates the response of apples to N deficiency.

Considering that the *MdATG8A* transcripts were degraded more slowly in OE2 than WT plants (Fig. 6e), we knocked down *MdATG8A* in OE2 (OE2-*MdATG8A*^Ri^) (Fig. S2, see online supplementary material). The production of biomass and relative autophagic activity had no significant difference between OE2-*MdATG8A*^Ri^ and WT (Fig. 7b, c, and d), which further indicated that MhYTP2 regulates the plant resistance to N deficiency through *MdATG8A.*

**Figure 8:**
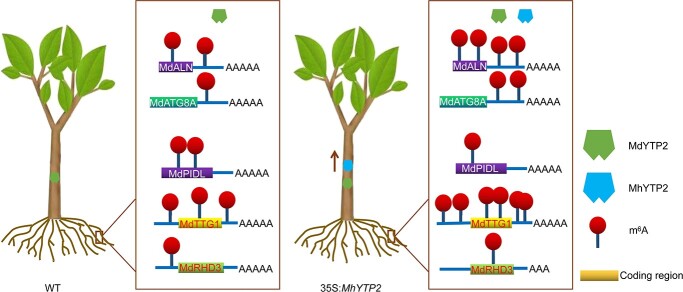
The proposed model describes how the m^6^A reader protein MhYTP2 regulates the stability of its target mRNAs that are related to low nitrogen tolerance.

These results collectively indicate that MhYTP2 binds with and stabilizes the mRNAs of *MdALN*, *MdPIDL*, *MdTTG1*, and *MdATG8A*, degrades the target *MdRHD3* mRNA, contributing to low N resistance in apple (Fig. 8).

## Discussion

Soil N availability usually limits plant growth and yield [26, 40, 41]. When there is little N in the soil, plants need to effectively recycle and recombine elements. The m^6^A modifications of RNA are increasingly significant mechanisms of plant resistance to starvation and stress. The *Arabidopsis* m^6^A reader CPSF30-L regulates nitrate signaling [42]. The apple m^6^A reader MhYTP2 responds to a variety of stress conditions, such as leaf senescence, extreme temperature, drought, high salinity, low nutrition, marssonina apple blotch, and powdery mildew pathogens inoculation [13, 23–25, 43]. In this study, we continued to investigate the biological function of MhYTP2 in N starvation. *MhYTP2* enhanced the resistance of apple to N deficiency. This study identified more biological functions of MhYTP2 in plants and revealed other potential roles in the regulation of mRNA stability.

Many organisms can use S-allantoin to make use of their stored N, carbon, and energy. Allantoin hydrolyzed by allantoase to allantoate is a common initial reaction of allantoin metabolism [44]. When the soil is poor in N, allantoin can be an alternative N source. Two functional allantoase genes, *AtALN* and *RpALN*, which are responsible for allantoin degradation, have been reported to be highly expressed in the absence of other N sources [45]. In the study, *MhYTP2* overexpressing lines had higher allantoinase activity than WT when plants were starved for N. Our analysis of *MdALN* expression showed that *MdALN* expression level in *MhYTP2* overexpressing lines was higher than WT under N deficit conditions. Under N starvation conditions, the allantoinase activity was higher in *MhYTP2* overexpressing plants which could degrade more allantoin to resist stress. To explore the potential mechanism, we analysed our previous m^6^A-seq data and found a significant induction of m^6^A modification for *MdALN* at the 3'UTR and exon regions in OE2 compared with WT plants (Fig. 2b). An EMSA analysis showed that MhYTP2 directly bound with the m^6^A-modified *MdALN* mRNA (Fig. S1, see online supplementary material)*.* The overexpression of *MhYTP2* reduced the degradation rates of the *MdALN* mRNA in OE2 (Fig. 2d). There were no significant differences in the biomass production and allantoinase activity with WT when *MdALN* was knocked down in OE2 (Fig. 3b), this suggests that MhYTP2 positively regulates tolerance against N starvation by increasing the activity of allantoinase.

Previous studies have indicated that lower levels of N can promote root growth [46]. In our experiment, N starvation promoted roots growth. Under the low N condition, total root length, surface area, volume, root tips and lateral roots number of *MhYTP2* overexpressing lines increased significantly (Fig. 4b), providing a basis for maximization using the N. The effect of N on root development is related to carbohydrate concentration and metabolism [47]. The results from our study showed that MhYTP2 could directly bind with and regulate the stability of m^6^A-modified mRNAs of *MdPIDL*, *MdTTG1*, and *MdRHD3*. To explore whether the role of MhYTP2 to resist low N was dependent on these mRNA targets, we knocked down *MdPIDL* and *MdTTG1* in OE2*.* It was discovered that the biomass production and root structure parameters did not differ significantly between OE2-*MdPIDL*^Ri^ and WT, as well as between OE2-*MdTTG1*^Ri^ and WT, suggesting that the MhYTP2 regulated the resistance to N deficiency in apple through *MdPIDL* and *MdTTG1* (Fig. 5a and b)*. MdRHD3* transcripts were degraded more rapidly in OE2 than WT (Fig. 4e). We overexpressed *MdRHD3* in OE2, no significant difference was found between OE2-*MdRHD3*^OE^ and WT in terms of the biomass production and root structure parameters (Fig. 5c), indicating that the function of MhYTP2 in regulating the resistance of apple to low N was also through *MdRHD3.* These data suggested that MhYTP2 positively regulates tolerance against N starvation by binding with its target mRNAs that are related to the development of root systems and controls their stability.

Autophagy facilitates the remobilization of nutrients under starvation condition [36]. *Arabidopsis atg* mutants are hypersensitive to N deficiency and are less efficient at remobilizing N [36, 48–50]. The rice (*Oryza sativa*) *Osatg7–1* mutant exhibited suppressed N remobilization [51]. The maize (*Zea mays*) *atg12* mutant seedlings exhibited impaired N remobilization under N starvation [52]. However, the heterologous overexpression of *ATG* genes in *Arabidopsis* increased its resistance to N starvation [37, 53–55]. These studies demonstrated the autophagy genes play a role in N remobilization under N starvation conditions. In addition, studies have shown that the epitranscriptome regulator YTHDF3 copes with nutrient deficiency by regulating autophagy [56]. Our data clearly indicated that the level of expression of *MdATG8A* was higher in *MhYTP2* overexpressing lines under N starvation condition (Fig. 6e), possibly because MhYTP2 enhances autophagy activity by binding with and stabilizing the *MdATG8A* mRNA. To explore whether the role of MhYTP2 to resist low N was dependent on *MdATG8A* mRNA, we knocked down the expression of *MdATG8A* in OE2*.* It was discovered that the biomass production and relative autophagic activity were no significant differences between OE2-*MdATG8A*^Ri^ and WT (Fig. 7b, c, and d), suggesting that MhYTP2 regulated the resistance to N deficiency in apple through *MdATG8A.* Therefore, in *MhYTP2* overexpressing lines, N and C are more efficiently recovered through autophagy, resulting in better growth of *MhYTP2* overexpressing plants under N starvation conditions.

Previous studies have found that the m^6^A in exon regions appears to destabilize the mRNAs, whereas m^6^A in UTR generally stabilizes mRNAs [25]. In this study, we found that the levels of *MdALN* modified by m^6^A were significantly increased at both the 3'UTR and exon regions in OE2 compared with WT plant (Fig. 2b), while there was an increase in the *MdALN* mRNAs in *MhYTP2* overexpressing plants compared with WT (Fig. 2c), which could suggest that modification by m^6^A in the UTR may have a greater effect on the stability of *MdALN* mRNA than that in the exon region in the growing stage of the plant in our study. The level of *MdPIDL* modified by m^6^A decreased in the exon region in OE2 with an increase in the stability of *MdPIDL* mRNA. The level of *MdTTG1* modified by m^6^A was slightly increased in the entire mRNA in OE2, and there was also an increase in the stability of *MdTTG1* mRNA. *MdRHD3* was modified by m^6^A in the 5'UTR in WT plant and in the exon region in OE2 plant, which led to the decreased stability of *MdRHD3* mRNA in OE2 plant (Fig. 4c and e). The *MdATG8A* mRNA contained significantly increased levels of m^6^A in their 3'UTR that resulted in enhanced corresponding stability of mRNAs in OE2 plant (Fig. 6c and e). All in all, the relationship between m^6^A distribution and mRNA stability is consistent with previous studies in apple [25, 57, 58]. However, the significant enrichment of m^6^A in the exon region was associated with the overall up-regulation of mRNA expression in *Arabidopsis* [20, 21]. These suggest that the relationship between m^6^A distribution and mRNA stability is different among species. The complex relationship in allopolyploid *Brassica napus* and its diploid progenitors depended on the presence or absence of m^6^A modification or the abundance of m^6^A modification [59].

This study provides information that can be used to introduce durable tolerance to N starvation in apple. Our findings provide new information for the post-transcriptional regulation of the N starvation response in apple, as well as a possible strategy to improve the resistance of apple to N starvation through genetic engineering in the future.

## Material and methods

### Treatment of apple plants

The WT and *MhYTP2* overexpressing *M. domestica* cv. ‘Roya Gala’ plants for N deficiency experiments were the same as those previously used in drought experiments [24]. Briefly, the plants were initially cultured on MS agar media for 40 days and then grown on rooting MS agar media for another 40 days. The plants were transplanted to small pots to test their responses to N depletion in a growth chamber after 60 days under long-day conditions (16 h light/8 h dark, 25°C).

The N deficiency experiments were conducted in the growth chamber above. Sand culture was performed by rinsing perlite with 0.1% (v/v) HCl and water as a substrate. The pots were evenly watered with Hoagland's nutrient solutions with varying concentrations of N (CK, 1 mM N and treatment, 0.1 mM), at a rate of 1000 mL every 7 days. The pH of nutrient solution was adjusted to 5.5. The pots were arranged completely at random. The plants were sampled after 0, 15, and 30 days of treatment. Ten leaves per plant line were harvested, frozen in liquid nitrogen, and stored at −80°C. The phenotypic record, stem lengths, SPAD values, allantoinase activity, allantoin concentration, and root system architecture were determined after 0 and 30 days of treatments. The levels of gene expression were determined in the samples collected.

### Physiological measurements

The SPAD values were determined using an SPAD-502 (Konika Minolta, Tokyo, Japan). The activity of allantoinase was measured spectrophotometrically using test kits (Sangon Biotech, Shanghai, China) according to the manufacturer's instructions.

The allantoin concentration was measured as previously described [60]. Allantoin is hydrolyzed by alkali-acid to glyoxylate. The glyoxylate is converted to glycoxylic acid phenylhydrazone, which is then oxidized to the red-colored 1, 5-diphenylformazan. The absorbance of the supernatant was measured using a spectrophotometer at 520 nm.

The root system architecture analysis was performed using a Winrhizo 2002 (Regent Corporation, Quebec, Canada) [61].

The autophagosomes were detected by a JEOL-1230 transmission electron microscope (Hitachi, Tokyo, Japan) [62].

### Generation of root transgenic apple plants

Root transgenic plants were generated using an *Agrobacterium*-mediated hairy root transgenic system as previously described [39]. WT and OE2 plants were initially cultured on MS subculture and rooting agar media for 40 days, respectively. The plants were then transplanted into small pots and maintained under long-day condition for 30 days. The *MdALN*-2300, *MdALN*-pK7, *MdPIDL*-2300, *MdPIDL*-pK7, *MdTTG1*–2300, *MdTTG1*-pK7, *MdRHD3*–2300, *MdRHD3*-pK7, *MdATG8A*-2300, and *MdATG8A*-pK7 plasmids were constructed. Then, the apple plants were infected with *Agrobacterium rhizogenes* K599 (Weidi Biotechnology, Shanghai, China) using empty vector 2300 or pK7 and constructed plasmids to generate transgenic roots.

### Quantitative real-time PCR

The RNA extraction using the plant RNA purification kit (Yeasen, Shanghai, China), and the first-strand cDNA synthesizing with a first strand cDNA synthesis master mix (Yeasen, Shanghai, China). Quantitative real-time PCR was performed on a LightCycler® 96 real-time PCR system (Roche, Basel, Switzerland) using a 2× ChamQ SYBR qPCR Master Mixture (Vazyme Biotech, Nanjing, China). The inner reference genes *MdActin* (XM_008344381) was used to normalize the genes expression using the 2^−∆∆Ct^ method [63, 64]. The primers are listed in Table S1 (see online supplementary material).

### mRNA stability assay

Tissue-cultured WT and OE2 plants were treated with modified MS agar with 0.2 mM actinomycin D. Tissues were collected at days 0, 5, 15, and 20 for transcription inhibition analysis as previously described [25].

### EMSA

An EMSA was performed as previously described [25]. The MhYTP2-His fusion protein was expressed and purified from *Escherichia coli* BL21. The digoxin-labeled RNA oligo-nucleotides for MhYTP2-His binding affinity assay were synthesized by Sangon Biotech (Sangon Biotech, Shanghai, China), which are listed in Text S2 (see online supplementary material). The concentration of RNA probe was 4 nmol and MhYTP2-His concentration ranged from 0 to 2000 nM.

### Statistical analysis

Statistical significance was determined using SPSS 21 (IBM, Inc., Armonk, NY, USA) and graphed using SigmaPlot 12.0 software (Systat Software, Inc., San Jose, CA, USA). The data were analysed using an independent *t* test (*P* < 0.05) or subjected to a one-way analysis of variance (ANOVA).

## Supplementary Material

Web_Material_uhad094

## Data Availability

All relevant data can be found within the paper and its supporting materials.
